# Practice Variation among Pediatric Endocrinologists in the Dosing of Glucocorticoids in Young Children with Congenital Adrenal Hyperplasia

**DOI:** 10.3390/children10121871

**Published:** 2023-11-29

**Authors:** Heba Al-Rayess, Amit Lahoti, Leslie Long Simpson, Elise Palzer, Paul Thornton, Ryan Heksch, Manmohan Kamboj, Takara Stanley, Molly O. Regelmann, Anshu Gupta, Vandana Raman, Shilpa Mehta, Mitchell E. Geffner, Kyriakie Sarafoglou

**Affiliations:** 1 Department of Pediatrics, Division of Endocrinology, University of Minnesota Medical School, Minneapolis, MN 55454, USA; alray007@umn.edu; 2 Department of Pediatrics, Division of Endocrinology, Nationwide Children’s Hospital at The Ohio State University, Columbus, OH 43205, USA; amit.lahoti@nationwidechildrens.org (A.L.); manmohan.kamboj@nationwidechildrens.org (M.K.); 3 Division of Biostatistics, University of Minnesota School of Public Health, Minneapolis, MN 55455, USA; longx006@umn.edu (L.L.S.); north266@umn.edu (E.P.); 4 Division of Endocrinology and Diabetes, Cook Children’s Medical Center, Fort Worth, TX 76104, USA; paul.thornton@cookchildrens.org; 5 Center for Diabetes and Endocrinology, Department of Pediatrics, Akron Children’s Hospital, Akron, OH 44308, USA; rheksch@akronchildrens.org; 6 Pediatric Endocrine Unit and Metabolism Unit, Massachusetts General Hospital and Harvard Medical School, Boston, MA 02114, USA; tstanley@mgh.harvard.edu; 7 Division of Pediatric Endocrinology and Diabetes, Children’s Hospital at Montefiore, Albert Einstein College of Medicine, Bronx, NY 10467, USA; moregelm@montefiore.org; 8 Division of Pediatric Endocrinology, Children’s Hospital of Richmond, Virginia Commonwealth University, Richmond, VA 23298, USA; anshu.gupta@vcuhealth.org; 9 Department of Pediatrics, Division of Pediatric Endocrinology, University of Utah, Salt Lake City, UT 84112, USA; vana.raman@hsc.utah.edu; 10 Department of Pediatrics, Division of Pediatric Endocrinology and Diabetes, New York Medical College, Valhalla, NY 10595, USA; 11 The Saban Research Institute, Children’s Hospital Los Angeles, The Keck School of Medicine of the University of Southern California, Los Angeles, CA 90033, USA; mgeffner@chla.usc.edu; 12 Department of Experimental and Clinical Pharmacology, University of Minnesota College of Pharmacy, Minneapolis, MN 55455, USA

**Keywords:** congenital adrenal hyperplasia, glucocorticoids, pediatrics, formulations, hydrocortisone

## Abstract

A Pediatric Endocrine Society (PES) Drugs and Therapeutics Committee workgroup sought to determine the prescribing practices of pediatric endocrinologists when treating children <10 years of age with congenital adrenal hyperplasia (CAH). Our workgroup administered a 32-question online survey to PES members. There were 187 respondents (88.9% attending physicians), mostly from university-affiliated clinics (~80%). Ninety-eight percent of respondents prescribed the short-acting glucocorticoid hydrocortisone to treat young children, as per the Endocrine Society CAH Guidelines, although respondents also prescribed long-acting glucocorticoids such as prednisolone suspension (12%), prednisone tablets (9%), and prednisone suspension (6%). Ninety-seven percent of respondents indicated that they were likely/very likely to prescribe hydrocortisone in a thrice-daily regimen, as per CAH Guidelines, although 19% were also likely to follow a twice-daily regimen. To achieve smaller doses, using a pill-cutter was the most frequent method recommended by providers to manipulate tablets (87.2%), followed by dissolving tablets in water (25.7%) to create a daily batch (43.7%) and/or dissolving a tablet for each dose (64.6%). Thirty-one percent of providers use pharmacy-compounded hydrocortisone suspension to achieve doses of <2.5 mg. Our survey shows that practices among providers in the dosing of young children with CAH vary greatly and sometimes fall outside of the CAH Guidelines—specifically when attempting to deliver lower, age-appropriate hydrocortisone doses.

## 1. Introduction

Young children with congenital adrenal hyperplasia (CAH) require smaller doses and incremental adjustments of glucocorticoids (GCs) to meet the goal of controlling excessive androgen production while also using the lowest possible doses to avoid the side effects of excess GCs. For growing children, hydrocortisone (HC), a short-acting GC, is recommended because long-acting GCs may have adverse effects on growth [1]. From 2001 until recently, the only commercially available formulation to treat children with CAH has been HC tablets containing 5 or 10 mg doses. If a pediatric endocrinologist wanted to make a small, incremental change in HC dosing of <2.5 mg, parents needed to split 5 mg tablets into quarters and sometimes even smaller pieces or manipulate tablets in other ways that could lead to inaccurate dosing and periods of hypo- and hypercortisolemia. A Pediatric Endocrine Society (PES) Drugs and Therapeutics Committee work group sought to determine the current prescribing practices of pediatric endocrinologists in treating infants and children with CAH < 10 years of age, with a specific focus on how incremental HC doses <2.5 mg are prescribed. A survey was made available to members of the PES in late fall 2020. At about the same time (on 29 September 2020), multi-particulate HC granules (Alkindi Sprinkle^®^) with doses of 0.5, 1, 2, and 5 mg were approved by the Food and Drug Administration (FDA) in the USA for use in young children with adrenocortical insufficiency [2]. Due to the concurrent timing of the PES survey administration with the FDA approval of HC granules, questions concerning *Alkindi Sprinkle^®^* were not included in the survey, as providers did not yet have experience using this formulation.

## 2. Materials and Methods

A working group from the PES Drugs and Therapeutics Committee developed a 32-question survey regarding which GCs, doses, and formulations were used in treating young children with CAH under the age of 10 years. The survey was reviewed and approved by the PES Research Affairs Committee and Board of Directors before being distributed to the PES membership. The survey was linked to REDCap and administered online to PES members between 21 October 2020 and 1 October 2021. All respondents were anonymous. Summary data were obtained by count and percent using R version 3.5.1 (R Foundation for Statistical Computing, Vienna, Austria). 

## 3. Results

The survey was sent out to 1598 PES members (83% attending physicians, 16% fellowship trainees, and 1% nurses). There were 187 respondents, the majority being attending physicians (88.9%) in university-affiliated clinics (~80%) who had practiced endocrinology for >5 years (72%). Over 50% were following more than six patients with CAH who were aged <10 years. Almost all respondents (97%) answered that they were very likely or likely to prescribe HC in thrice-daily doses. Interestingly, 19% of participants were equally likely to prescribe a twice- or thrice-daily regimen of HC. Most respondents reported using HC doses in the recommended range of 8–14.9 mg/m^2^/day [1]. 

**Figure 1:**
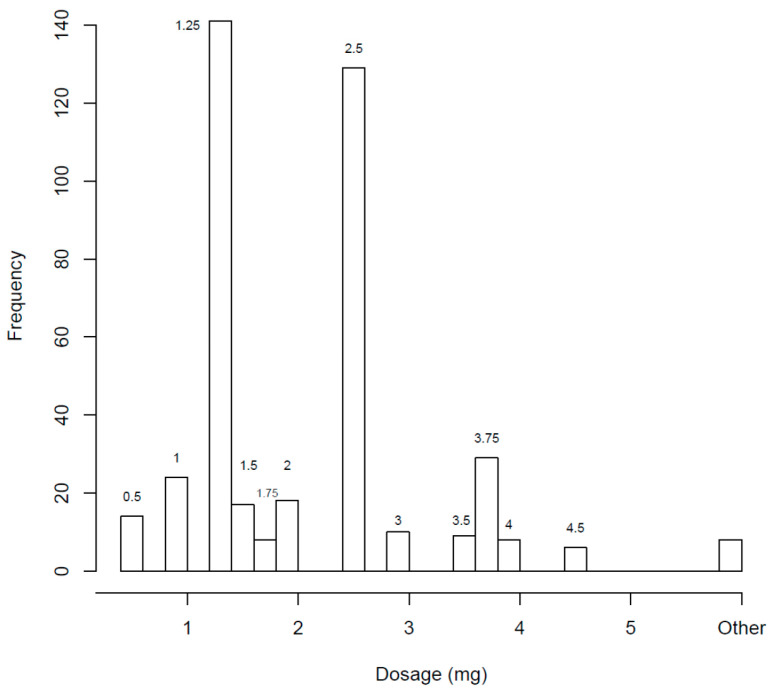
Frequency with which providers (n = 184) prescribed individual hydrocortisone tablet doses in increments of <5 mg to treat children with CAH < 10 years of age. * Respondents were asked to check all doses that they have used in their practice to treat children with CAH < 10 years of age. Frequency is the number of providers who have used each of the doses.

Table 1 shows the types of GCs prescribed. When asked about HC doses < 5 mg that they had prescribed in their practice, 69% responded “Yes” to prescribing 2.5 mg and 75% to prescribing 1.25 mg doses. Figure 1 shows the most commonly prescribed individual doses when using tablets after manipulation. 

Table 2 shows the methods used to manipulate HC tablets, with a pill-cutter being the most frequent, followed by dissolving tablets in water. When dissolving tablets, providers instructed parents to create a daily batch (43.7%) and to dissolve a tablet for each dose (64.6%), with some providers recommending both methods. About 8.3% of providers did not know how their patients created the dissolved solution. Of the providers who prescribed a HC suspension (n = 58), 24% used an alcohol-free suspension, 22% hydrocortisone succinate, 38% used other compounded formulations, and 14% were not sure what suspension was used. 

**Figure 2:**
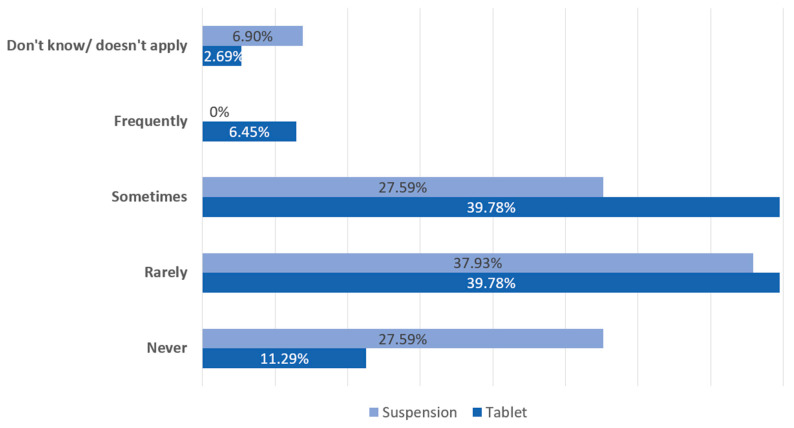
How often do you receive complaints or comments from your patients’ families about difficulty in dividing tablets vs. difficulty using suspension?

Figure 2 shows that physicians received more complaints from parents about the challenges of achieving smaller doses from HC tablets than from HC suspensions. Table 3 lists the reasons that physicians did not prescribe pharmacy-compounded HC suspensions. The most cited reasons for not using pharmacy-compounded liquid formulations, even though they would allow dosing in increments as low as 0.5 mg, were that they were taught not to do this during fellowship training (n = 87), CAH consensus guidelines (n = 81), and not having a reliable compounding pharmacy (n = 50). Nonetheless, 63% of respondents indicated that they would prescribe HC suspension if it were commercially available. Side effects of over- or under-treatment were reported to be less frequently observed when prescribing pharmacy-compounded HC suspensions (10%) compared to dividing tablets (18%) to achieve HC doses of <2.5 mg. 

## 4. Discussion

Until recently, most commercially available GC formulations have been geared towards treating adults and are not designed to allow for small, incremental dosing of <2.5 mg of HC in infants, toddlers, and young children. Our report is the first to show the practice variability in the delivery methods, regimens, and type of GCs used in treating young children with CAH in the USA and highlights the importance of developing age-appropriate HC formulations.

At the time that the survey was administered, the only HC formulation with which pediatric endocrinologists had experience was HC in 5 and 10 mg scored tablets. Most of the providers used scored 5 mg tablets to deliver small doses ranging from 0.5 to 4.5 mg (n = 184/187), which required splitting the 5 mg tablets more than once, followed by the administration of pharmacy-compounded suspensions (n = 58). 

Hydrocortisone cypionate suspension was commercially available in the United States until a study in 2001 [3] raised appropriate concerns about its lower cortisol bioavailability compared to tablets. One unfortunate consequence of this study has been the misinterpretation that all suspension formulations have poor cortisol bioavailability, even though the article stated that studies of other non-commercial formulations have shown stability and dosage uniformity [4,5]. A more recent study compared cortisol exposure and pharmacodynamic adrenal steroid responses to a pharmacy-compounded alcohol-free HC suspension versus commercially available HC tablets and found no significant differences in cortisol pharmacokinetic/pharmacodynamic parameters between the two formulations [6]. In another study comparing epiphyseal (bone age) maturation and growth outcomes, treatment with HC suspension decreased androgen exposure, as shown by lower bone-age z-scores, and allowed a lower average and cumulative daily HC dose compared to HC tablets [7]. More than half of our survey respondents would use HC suspension if it were commercially available, although about one-third of the participants were unsure if they would. Table 4 lists multiple studies of liquid HC formulations that have shown acceptable uniformity and stability. 

Since the 2001 study [3] and until September 2020, the only commercially available formulations to treat children with CAH were scored HC tablets with 5 and 10 mg doses. When a dose <2.5 mg is required, parents must split 5 mg tablets into quarters or smaller pieces. Splitting a HC tablet into quarters may result in dosing outside of the targeted dose range [15,16], which may have significant consequences in young children such as iatrogenic Cushing’s syndrome or failure to thrive, or recurrent hypoglycemia [17,18]. Another study found that tablets should not be split more than once due to uncertainty in dose accuracy and concluded that there is a need for commercially available age-appropriate formulations [19]. Additionally, splitting a tablet more than once to achieve doses <2.5 mg is not FDA-approved.

Based on our survey results, pediatric endocrinologists use a variety of GCs, formulations, and manipulation methods to administer small doses (<2.5 mg) in young children with CAH. For example, despite the consensus statement recommendation against using longer-acting GCs to treat CAH and other forms of primary adrenal insufficiency in growing children [1], our survey found that prednisolone suspension (12%) and prednisone tablets (9%) were still reported as the prescribed formulations for children with CAH < 10 years of age. Oral prednisone and prednisolone are 4–5 times more potent than HC, and dexamethasone is 50–100 times more potent than HC [20,21]. While our survey did not assess reasons for this usage pattern, potential explanations for using long-acting GCs could be suboptimal disease control due to poor compliance with thrice-daily hydrocortisone dosing. 

Regarding HC tablets, 75% of respondents have prescribed a dose of 1.25 mg (a quartered 5 mg tablet). The most commonly recommended method to divide tablets was using a pill-cutter, which has been shown to be a more accurate way of splitting tablets than by hand [22]. However, quartering single-scored tablets has been shown to produce inaccurate dosing. Andersson et al. reported that halved HC tablets passed the European Pharmacopoeia criteria for weight variability, but once quartered, all of the tablets were outside the expected range for weight [19]. Besides confirming the weight variability of quartered HC tablets, another study found that HC content was correlated with quartered tablet weight and that 54% of the quartered 10 mg HC tablets failed to achieve ±10% of the target dose [15]. Cutting HC tablets to achieve a prescribed dose might produce small fragments, even smaller than a quarter. In a previously published survey, caregivers for children under 6 years of age reported that >50% of the prescribed doses could not be prepared by simply halving or quartering the tablets [23]. In our survey, doses of 0.5 mg, 1 mg, 1.5 mg, 2 mg, 3 mg, 3.5 mg, 4 mg, and 4.5 mg were prescribed by clinicians, but it was not specified whether they were cut or dissolved in water.

Our survey also found that the second most popular method to obtain prescribed doses of <2.5 mg was the dispersion of tablets in water to create a solution and then drawing the prescribed dose over the course the day, which was recommended by 25% of our respondents. When dissolving HC, one might assume that the medication is equally distributed in the solution. However, this is not the case, as HC is hydrophobic. This would result in the child receiving significantly varying doses, either more or less than the prescribed dose. An experiment dispersing tablets of aspirin, which is also hydrophobic, found that obtaining the target dose varied greatly depending on which zone in the container the intended dose was drawn (e.g., the base of the container (zone 1) or the top of the solution (zone 5)) [24]. Doses larger than intended were drawn from the second zone from the bottom (zone 2), sometimes reaching double the required dose. When the dose was obtained from higher zones, the extracted dose decreased, and the mean obtained dose from the top zone was only 35–50% of the intended dose [24]. 

CAH consensus guidelines state that “Good control can be achieved by orally administering crushed, weighed hydrocortisone tablets mixed with a small volume of liquid, if needed, immediately before administration” [1]. This statement meant to suggest that the intended dose of HC can be crushed and mixed in liquid, and then taken all at once for younger children and individuals who are averse to swallowing tablets. This statement is, however, sometimes misinterpreted to mean that mixing tablets in liquid to create a daily batch to draw individual doses over 24 h can be an acceptable way of administering doses <2.5 mg. It has been reported that, for children whose parents administer 10 mg HC tablets dispersed in water, the dose that the children received was outside the ±20% range of the target dose more than half of the time, with some individual doses >250% of the desired target dose [23]. Nearly half of our survey respondents who advised dissolving tablets recommended doing so by creating a single batch to use for the day. This could theoretically cause even more variability in dosing if the drug particles were more sedimented in the deeper areas. Iatrogenic Cushing’s syndrome has been reported when dispersing HC tablets in water [17] and when compounded into capsules [25]. Al-Rayess et al. described a 6-year-old girl who developed iatrogenic Cushing’s syndrome due to overtreatment when parents dissolved 10 mg HC tablets in water as a daily batch and drew individual doses across the day from that batch. The Cushing’s syndrome symptoms resolved after she was switched to a pharmacy-compounded suspension at a similar dose [17]. Another study tested variability in HC doses by examining medication amounts delivered through a nasogastric tube. The dose was prepared by crushing and dispersing either HC suspension or HC granules [26]. Crushed and dispersed HC tablets given through 12-French nasogastric tube resulted in a mean dose of 174% of the target dose.

A limitation of this report is that the survey was conducted just as HC granules (Alkindi Sprinkle^®^*)* were approved by the FDA, so we were not able to collect provider feedback on its use. Of note, while HC granules allow for accurate low-dosing of HC (0.5, 1, 2, and 5 mg), its use has not been universally accepted by insurance companies, with prescriptions often requiring appeals despite the fact that they are the only FDA-approved way of administering doses other than in 2.5 mg increments. A follow-up survey of pediatric endocrinologists to inquire whether HC granules have addressed the gap of delivering small, incremental doses <2.5 mg after they have been on the market for 5 years is warranted. That said, the value of this report comes from illustrating, in detail, the practice variation between pediatric endocrinologists. As reported, some pediatric endocrinologists are still prescribing long-acting glucocorticoids, despite the Endocrine Society guidelines recommending against their use in most pediatric cases. Additionally, pediatric endocrinologists use different methods of manipulation that may not deliver accurate dosing. Our report emphasizes the importance of giving small doses and the minimization of the manipulation of tablets by parents. Reports such as ours should actually help with insurance coverage of current and potentially new pediatric formulations of HC that allow for small and accurate dose increments. Some areas being explored are commercially available alcohol-free HC suspension and point-of-care 3D-printed immediate- and sustained-release tablets for the personalized treatment of children with CAH and other forms of AI [27,28,29]. 

## Figures and Tables

**Table 1:** Glucocorticoid formulations used to treat children with CAH <10 years of age.

GC Formulations	Yes *	No
Hydrocortisone tablets	184	3
Hydrocortisone suspension	58	129
Dexamethasone tablets	5	182
Dexamethasone suspension	3	184
Prednisone tablets	16	171
Prednisone suspension	12	175
Prednisolone tablets	5	182
Prednisolone suspension	22	165

* Respondents were asked to “check all that apply” to their practice; hence, totals exceed the number of respondents. GC, glucocorticoids.

**Table 2:** Methods of tablet manipulation used to achieve hydrocortisone doses of <2.5 mg.

Methods to Achieve Doses of <2.5 mg	Count	Percent *
Cut by hand	20	10.7
Use a pill-cutter	163	87.2
Use a knife	23	12.3
Dissolve tablets in water	48	25.7
Does not apply to my practice	4	2.14
Don’t know	3	1.6
Other	9	4.81

* Respondents were asked to “check all that apply” to their practice; hence, total percentage exceeds 100.

**Table 3:** Reasons reported for not using a pharmacy-compounded HC suspension.

Reason	Count	Percent *
Associated with overtreatment	15	11.6
Associated with undertreatment	21	16.3
Hydrocortisone suspension not available	14	10.8
No reliable compounding pharmacy	50	38.7
CAH consensus guidelines	81	62.7
Insurance won’t cover	10	7.7
My training	87	67
Other	12	9.3

* Respondents were asked to “check all that apply” to their practice; hence, total percentage exceeds 100. HC, hydrocortisone.

**Table 4:** Hydrocortisone suspension formulations.

Author, Year (Citation)	Suspension Components	Concentration	Outcomes
Fawcett 1995 [4]	Hydrocortisone 20 mg tablets or powder Polysorbate 80 Sodium carboxymethylcellulose syrup BP methyl- and propyl-hydroxybenzoate citric acid monohydrate water	2.5 mg/mL	- Stable at 5 °C and 25 °C for 90 days - Dose-uniformity confirmed. - Maximum stability at pH 3–4 - More stable if prepared from powder compared to tablets
Chong et al., 2003 [8]	1:1 mixture of Ora-Sweet and Ora-Plus	1 mg/mL and 2 mg/mL	Physically and chemically stable for up to 91 days at 4 °C and 25 °C
Allen 2004 [9]	100-mg hydrocortisone powder (5 crushed 20 mg tablets) Ora-Plus Plus (suspending agent) 45 mL Ora-Sweet or Ora-Sweet SF (flavoring agent) 100 mL Glycerin 5 mL	2 mg/mL	No outcomes studied
Gupta 2007 [10]	Ethyl alcohol Hydrocortisone Glycerin Ora-Sweet Humco simple syrup Water	2 mg/mL	Stable for at least 60 days when stored in amber-colored glass bottles at room temperature
Santovena 2010 [11]	Hydrocortisone Carboxymethylcellulose Polysorbate-80 Methyl-p-hydroxybenzoate Propyl-p-hydroxybenzoate Sucrose Citric acid Purified water Syrup	1 mg/mL	Stability confirmed for 90 days at 5 °C
Orlu-Gul 2013 [12]	Citric acid buffer (pH 4.2) Hydroxypropyl B-cyclodextrin Orange tangerine (flavoring) Methyl paraben sodium salt/potassium sorbate (preservative) Neotame 0.075% sweetener	5 mg/mL	Stable for 28 days in refrigerator or at room temperature
Chappe 2015 [13]	Hydrocortisone succinate powder in citrate buffers or with sterile water	1 mg/mL	Stable for 14 days only with refrigeration
Manchanda 2018 [14]	10-mg tablets in a dye-free oral vehicle (Oral Mix, Medisca).	2 mg/mL	Solubility 230 mcg/mL Stable for 90 days at 4 °C and 25 °C

BP, British Pharmacopeia; SF, Sugar-free.

## Data Availability

Data are contained within the article.
